# Distinct Neuroblastoma-associated Alterations of *PHOX2B* Impair Sympathetic Neuronal Differentiation in Zebrafish Models

**DOI:** 10.1371/journal.pgen.1003533

**Published:** 2013-06-06

**Authors:** Desheng Pei, William Luther, Wenchao Wang, Barry H. Paw, Rodney A. Stewart, Rani E. George

**Affiliations:** 1 Department of Pediatric Oncology, Dana-Farber Cancer Institute, Division of Hematology/Oncology, Boston Children's Hospital, Harvard Medical School, Boston, Massachusetts, United States of America; 2 Division of Hematology, Brigham & Women's Hospital, Harvard Medical School, Boston, Massachusetts, United States of America; 3 Department of Oncological Sciences, Huntsman Cancer Institute, University of Utah, Salt Lake City, Utah, United States of America; University of Washington, United States of America

## Abstract

Heterozygous germline mutations and deletions in *PHOX2B*, a key regulator of autonomic neuron development, predispose to neuroblastoma, a tumor of the peripheral sympathetic nervous system. To gain insight into the oncogenic mechanisms engaged by these changes, we used zebrafish models to study the functional consequences of aberrant *PHOX2B* expression in the cells of the developing sympathetic nervous system. Allelic deficiency, modeled by *phox2b* morpholino knockdown, led to a decrease in the terminal differentiation markers *th* and *dbh* in sympathetic ganglion cells. The same effect was seen on overexpression of two distinct neuroblastoma-associated frameshift mutations, *676delG* and *K155X* - but not the *R100L* missense mutation - in the presence of endogenous Phox2b, pointing to their dominant-negative effects. We demonstrate that Phox2b is capable of regulating itself as well as *ascl1*, and that *phox2b* deficiency uncouples this autoregulatory mechanism, leading to inhibition of sympathetic neuron differentiation. This effect on terminal differentiation is associated with an increased number of *phox2b^+^*, *ascl1^+^*, *elavl3^−^* cells that respond poorly to retinoic acid. These findings suggest that a reduced dosage of *PHOX2B* during development, through either a heterozygous deletion or dominant-negative mutation, imposes a block in the differentiation of sympathetic neuronal precursors, resulting in a cell population that is likely to be susceptible to secondary transforming events.

## Author Summary

Neuroblastoma, a tumor of the peripheral sympathetic nervous system, is the most common cancer diagnosed in infancy. Although most cases arise sporadically, familial predisposition also occurs in association with mutations in a single copy of the *PHOX2B* gene, a “master regulator” of sympathetic neuronal development. The exact mechanisms by which these mutations increase susceptibility to neuroblastoma are unclear, primarily because of the paucity of optimal models in which to study very early development of the sympathetic nervous system. We took advantage of the ex vivo development and transparent nature of zebrafish embryos to study the roles of both normal and mutated PHOX2B in development of the sympathetic nervous system. We present data indicating that aberrant PHOX2B expression causes an arrest in the normal maturation of sympathetic neurons, leading to immature cells that are resistant to drug-induced differentiation. Indeed, we demonstrate that *phox2b* gene “dosage” is important for normal differentiation of sympathetic neurons in the zebrafish and suggest that the population of immature cells resulting from a decreased dosage of this pivotal factor may be susceptible to secondary mutations that could ultimately lead to neuroblastoma.

## Introduction

Neuroblastoma is an embryonal malignancy of the peripheral sympathetic nervous system (PSNS) that arises from the developing neural crest and manifests as neoplasms in sympathetic ganglia or adrenal medulla. The oncogenic events culminating in neuroblastoma are thought to occur very early in development, consistent with the status of this tumor as the most common cancer of infants [1]. A defining feature of neuroblastic tumors is their broad spectrum of cellular differentiation, ranging from undifferentiated cells that indicate a poor prognosis to those showing greater differentiation and predicting a generally favorable outcome [2]. This heterogeneity suggests that dysregulated differentiation of sympathetic progenitor cells plays a key role in neuroblastoma pathogenesis. Direct evidence for this model comes from the identification of heterozygous germline mutations in the homeodomain transcription factor *PHOX2B*, a regulator of sympathetic neuronal differentiation [3]–[8]. Such mutations are also found in patients with other neural crest-derived disorders, including congenital central hypoventilation syndrome (CCHS) and Hirschprung's disease, characterized by absent or abnormal development of the noradrenergic neurons in the brain stem and colon, respectively [3], [5], [7], [8].

The vast majority of embryonal tumors like neuroblastoma arise from aberrant genetic and epigenetic changes that control the survival, proliferation and differentiation of specific tissues. Hence, one way to decipher the tumorigenic contribution of germline changes in highly conserved genes such as *PHOX2B* is to understand how they perturb normal development. The sympathetic nervous system is derived from the neural crest, a multipotent embryonic structure consisting of a transient population of cells that migrate from the neural tube to the region of the dorsal aorta during development [9]–[11]. At the dorsal aorta, prespecified SOX10-positive sympathetic progenitors, under the influence of bone morphogenetic proteins (BMPs), start the process of differentiation into noradrenergic neurons [12]. *Phox2b* and the basic helix-loop-helix (bHLH) factor *Ascl1* are the first transcription factors that appear upon initiation of differentiation of sympathoadrenal precursors [13]–[15]. *Phox2a* is expressed downstream of both *Phoxb* and *Ascl1* [13], [16], [17] but its role in the initial stages of the sympathetic lineage remains undefined. Further neuronal differentiation occurs under the influence of the transcription factors *Hand2* [18], [19], *Gata2/3* [20] and *Tfap2a* [21], [22], which interact in a complex regulatory network to ultimately induce the expression of tyrosine hydroxylase (*Th*) and dopamine beta hydroxylase (*Dbh*) - two enzymes required for catecholamine production that serve as markers of terminal sympathetic neuronal differentiation [23]–[25]

Extensive work in murine and avian models has established *Phox2b* as a key regulator of autonomic neuron development, as its complete absence leads to embryonic lethality in mice due to the failure of sympathetic nervous system formation [15], [26]. Phox2b is first expressed in the murine peripheral sympathetic primordial at the dorsal aorta at E10.5 [13], [27]. By E13.5, it is expressed in all sympathetic progenitor cells including those of the superior cervical ganglia, paravertebral and pelvic ganglia and the adrenal medulla [27], and expression continues into the early postnatal period [28]. As the ganglia/adrenal medullae mature and the sympathoblasts differentiate, TH- and DBH-expression increases while PHOX2B expression decreases, so that by P28, only 12% of TH-positive sympathetic neurons have PHOX2B expression (compared to approximately 60% at P4) [28]. Hence, *Phox2b* is downregulated during terminal neuronal differentiation [28]. *Phox2b* also has growth inhibitory effects, as its overexpression promotes cell cycle exit and inhibits the proliferation of cultured sympathetic neurons [29], [30]. Most of the activity of the *Phox2b* promoter depends on an autoregulatory loop enabling the gene to regulate its own activity and that of other genes [31].

The human *PHOX2B* gene maps to chromosome 4p13 and consists of three exons encoding a highly conserved 314–amino-acid protein with two polyalanine repeats of 9 and 20 residues C-terminal to the homeodomain. Unlike the case in CCHS, which is defined largely by polyalanine repeat expansion mutations that lead to expansion of the second polyalanine tract [8], [32], [33], germline *PHOX2B* mutations associated with neuroblastoma tend to be (i) missense alterations in highly conserved regions [4], [7], [33] or (ii) mutations that result in a frameshift, giving rise to an altered or truncated protein lacking the second polyalanine motif [5], [8], [33], [34]. More recently, whole-allele deletions resulting in the reduction of the protein have been reported [35]. Still, the mechanisms by which these different classes of alterations predispose individuals to tumor development in the PSNS remain somewhat unclear. Partial loss of function with the preserved ability to suppress cellular proliferation but not to promote differentiation [36], complete loss of function due to functional haploinsufficiency [37] and both dominant-negative and gain-of-function effects [30], [34], [38] have all been proposed as contributors to neuroblastoma predisposition. In the single in vivo study to date, heterozygous insertion of two frameshift variants, *931del5* and *693del8*, in the mouse *Phox2b* locus resulted in impaired proliferation of sympathetic ganglion progenitors and biased differentiation towards the glial lineage, leading the authors to conclude that these mutants exhibited both dominant-negative and gain-of-function effects [38].

In this study we sought to analyze the effects of aberrant *PHOX2B* expression on sympathetic neuron development for each of the major classes of neuroblastoma-associated mutants. We selected the zebrafish model for this purpose because its development occurs ex utero, and the embryos survive considerably longer than mouse embryos, permitting analysis of the PSNS at later stages of sympathetic neuron differentiation and maintenance [39]. We show here that allelic *PHOX2B* deletion, modeled by morpholino (MO) knockdown, leads to a decrease in sympathetic neuronal differentiation in the PSNS. A similar loss of differentiation was observed upon overexpression of a neuroblastoma-linked truncation mutation *(K155X)* [8] and a frameshift mutation *(676delG)* [5] in the presence of endogenous *phox2b*, indicating that these variants function in a dominant-negative manner. By contrast, the *R100L* missense mutation [7] lacked any discernible effect on sympathetic ganglion development in the zebrafish embryo. We demonstrate further that the decrease in terminal differentiation was associated with an increased number of undifferentiated sympathetic neuronal precursors that were resistant to the effects of retinoic acid (RA), and generated a pool of developmentally arrested cells that could serve as targets for future transforming events.

## Results

### *phox2b* is expressed in cells of the PSNS in the zebrafish

**Figure 1:**
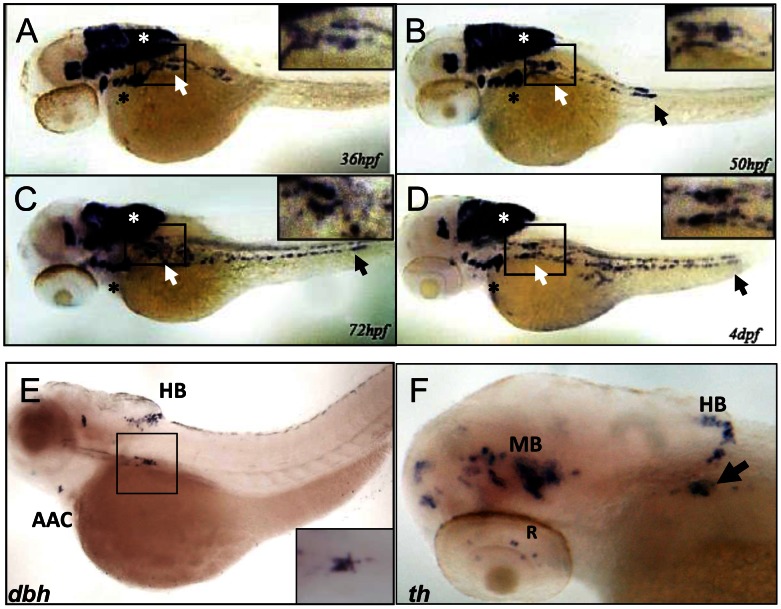
*phox2b* is expressed in the SCG, a marker of the peripheral sympathetic nervous system in the zebrafish. (A–D) Whole-mount *in situ* hybridization (ISH) for *phox2b* expression in wild-type embryos at the indicated time points. *phox2b* expression is seen in cells of the prospective superior cervical ganglion (SCG; white arrows, boxed) at 36 hpf (A), which start to extend caudally to form the sympathetic chain. These cells are identified as sympathetic neuronal cells by their expression of *dbh* and *th* (E, F). Expression is also seen in the brachial arches (black asterisk) and hindbrain (white asterisk). By 50 hpf, *phox2b* expression is seen in the enteric precursors (B–D, black arrow). Expression continues in two parallel rows caudally, encompassing the sympathetic chain until 4 dpf. (E) Lateral view (cranial to the left) of a 4-dpf zebrafish embryo analyzed by whole-mount ISH for *dbh* expression, which is seen in the SCG (black square), the arch associated complex (AAC) also derived from the neural crest, and the hindbrain (HB). Inset shows enlarged dorsal view of the SCG. (F) Whole-mount ISH of 4-dpf embryo analyzed for *th* expression. Aggregates of cells expressing *th* are seen in the SCG (arrow) as well as in the midbrain (MB), HB and retina (R).

To establish the zebrafish as a model for studying PHOX2B function in PSNS development, we analyzed expression of the *phox2b* gene in the superior cervical ganglion (SCG). The SCG is the earliest sympathetic ganglion to develop, starting at about 36 hours postfertilization (hpf) and progressing in size until about 4 days postfertilization (dpf) (Figure 1A–1D) [18], [21], [39], [40]. SCG cells are located ventral to the notochord between somites 1 and 4 and are easily visualized as several smaller cell aggregates that progressively increase in size, ultimately forming two separate ganglia in an hourglass shape. *phox2b* expression is first seen in the SCG cells at 36 hpf extending caudally in two irregular parallel rows to form the primary sympathetic ganglion chain to somite 11 (Figure 1A–1D). It remains robust in the SCG until 72 hpf (Figure 1C) when it gradually begins to be replaced by increasing numbers of cells expressing *dbh* and *th*, markers of terminal differentiation (Figures 1E–1F), whose expression is first seen at about 48hpf. This expression pattern, especially in the peripheral sympathetic ganglia, and the close sequence homology with human PHOX2B [41] (Figure S1) support the use of the zebrafish to model PHOX2B function in the PSNS.

### *phox2b* deficiency leads to decreased PSNS differentiation

**Figure 2:**
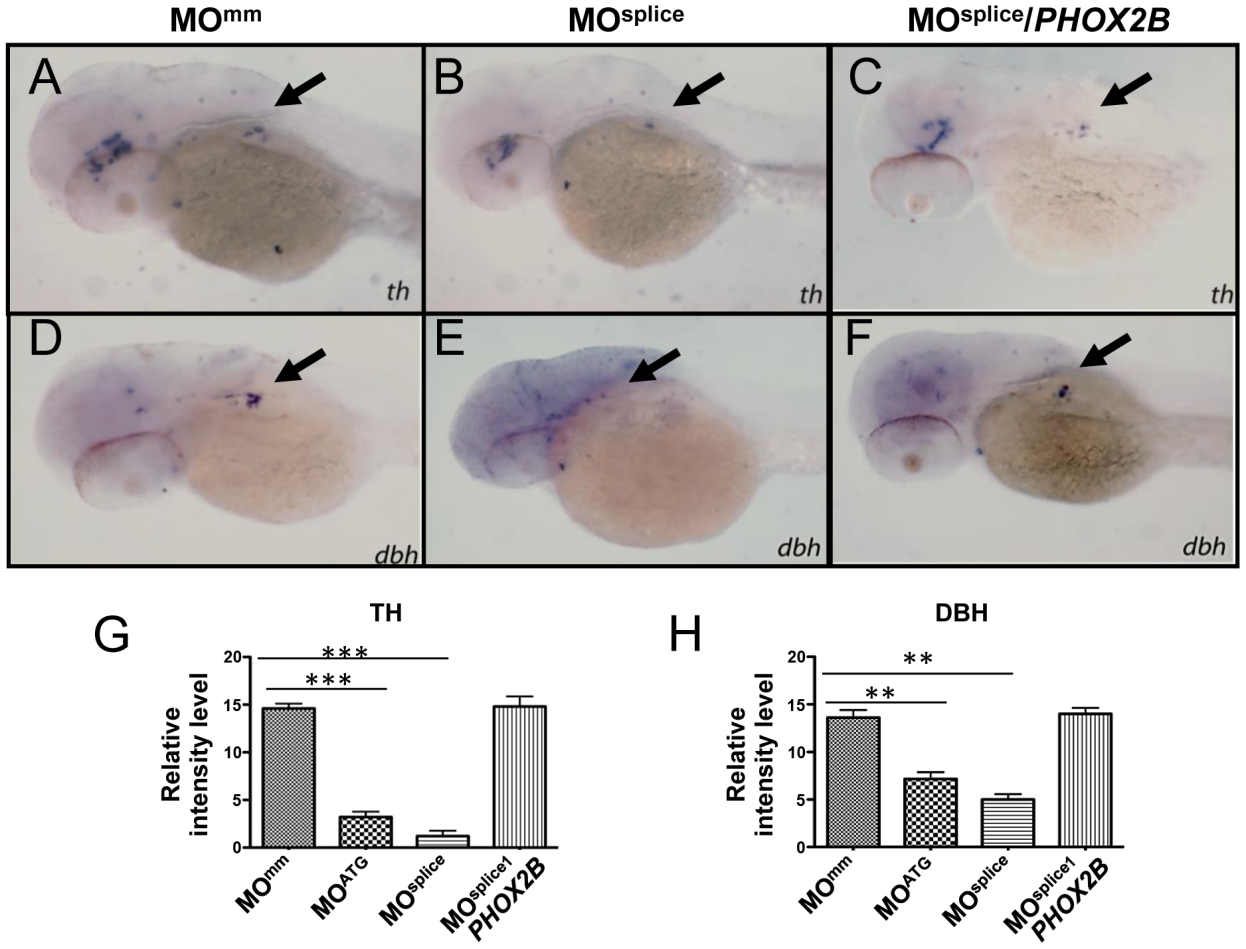
*phox2b-*deficient embryos show impaired differentiation of sympathetic neurons in the SCG. (A–F) Lateral/oblique views of 4-dpf embryos after whole-mount ISH for *th* (A–C) and *dbh* (D–F) in control and *phox2b*–deficient embryos. Arrows indicate the SCG. Knockdown of *phox2b* by injection of a splice MO (4 ng) (MO^splice^) inhibits the expression of *th* and *dbh* (B, E) which is rescued by coexpression of human *PHOX2B* mRNA (10 ng/µl) (C, F). Relative intensity levels of *th* (G) and *dbh* (H) expression in embryos injected with *phox2b* MOs that inhibit translation (MO^ATG^) or splicing (MO^splice^). Mismatched control MO (MO^mm^) and *PHOX2B* mRNA-rescue (MO^splice^/*PHOX2B*) are also shown. Data are presented as means ± SD (^***^P<0.001; ^**^P<0.01; n = 15 for each group).

To study the consequences of allelic *PHOX2B* deletion in patients with neuroblastoma [35], we performed morpholino (MO) knockdown of zebrafish *phox2b*, using two non-overlapping antisense oligonucleotide sequences targeted to the *phox2b* gene: a translation-blocking MO (MO^ATG^) and a splice-blocking MO (MO^splice^) directed to the second exon/intron splice junction (Figure S2A). *phox2b* knockdown was confirmed by immunoblotting in the case of the ATG MO (which showed ∼70–80% knockdown) and RT-PCR for the splice MO (Figure S2B). Experiments were performed in both wild-type and *p53* mutant embryos (*tp53^M214K/M214K^*) [42] to account for potential nonspecific effects associated with some MO injections, with similar results obtained in both backgrounds (Figure S2C). MO knockdown of *phox2b* led to a marked reduction in *th* and *dbh* expression in the SCG at 3 dpf, as compared with mismatched control MO-injected (MO^MM^) or uninjected wild-type (WT) siblings (Figures S3A, S3B, S3D, S3E, S3G, S3H). This phenotype was consistent with both the ATG and the splice MOs. To ensure that the decrease in *th* and *dbh* was not secondary to a general delay in development due to MO injection, we examined the SCG at 4 dpf (Figures 2A, 2B, 2D, 2E, 2G, 2H) and later (5 dpf; data not shown), again noting a decrease in the expression of these genes. To confirm that the phenotype was specific to *phox2b*, we rescued the *th-* and *dbh-*expressing cells by coexpressing human *PHOX2B* mRNA with both MOs, which led to an increase in *th* and *dbh* expression in the SCG (Figures 2C, 2E; 2G, 2H; Figures S3C, S3F; 3G, 3H). These results indicate that *PHOX2B* is necessary and sufficient for the terminal differentiation of sympathetic neuronal precursors.

### The *676delG* and *K155X* variants cause a decrease in terminal differentiation of sympathetic progenitors

**Figure 3:**
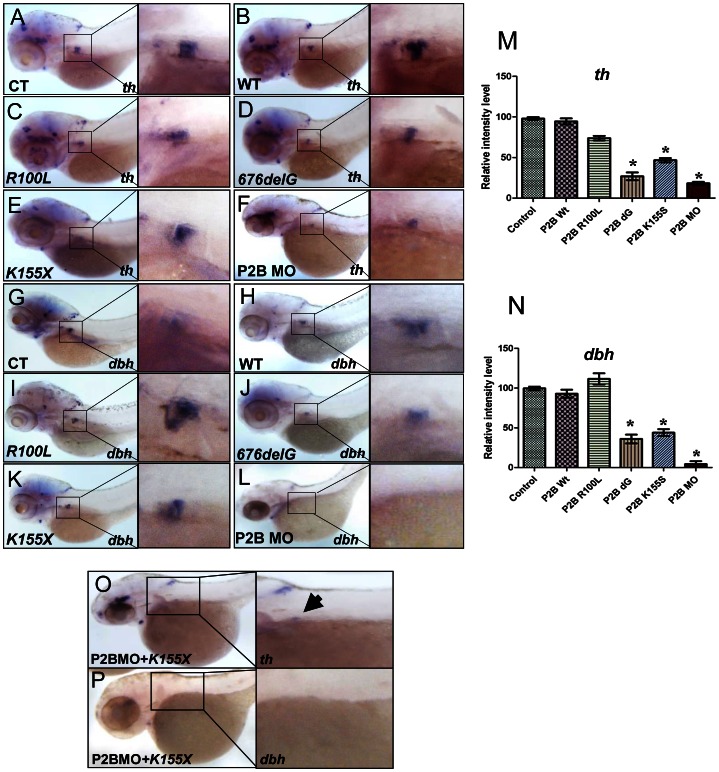
The neuroblastoma-associated *676delG* and *K155X PHOX2B* variants cause decreased terminal differentiation in the SCG. Whole-mount ISH for *th* (A–F) and *dbh* (G–L) expression in 3-dpf embryos in which human neuroblastoma-derived mutations were overexpressed (lateral views are shown). The area encompassing the SCG (boxed) is shown enlarged to the right of each panel. Capped mRNA (100 ng/µl) for wild-type (WT) human *PHOX2B* and the *R100L*, *676delG*, *K155X* mutations were injected into one-cell embryos. CT, control water-injected. Relative intensity levels of *th* (M) and *dbh* (N) expression in the embryos depicted in panels A–F and G–L respectively. Data are presented as means ± SD (*P<0.01 vs. control-injected embryos; n = 6 per group). Whole-mount ISH for *th* (O) and *dbh* (P) in 3-dpf embryos expressing the *phox2b* MO and *PHOX2B K155X* mutant mRNA (P2BMO+*K155X*). Arrow indicates the region of the SCG.

We next examined the effects of three distinct neuroblastoma-associated *PHOX2B* mutations on PSNS development (Figure S4, Figure 3). Overexpression of the *676delG* frameshift [5] and *K155X* truncation [8] variants led to a significant decrease in the expression of *th* (Figure 3D, 3E, 3M) and *dbh* in the SCG (Figure 3J, 3K, 3N) as compared to that in control (Figure 3A, 3G) and WT (Figure 3B, 3H) *phox2b* RNA-injected animals, but had a similar effect to MO knockdown (Figure 3F, 3L). By contrast, overexpression of the *R100L* homeodomain missense mutation [7] did not lead to a discernible change in the expression of either *th* or *dbh* in the SCG (Figure 3C, 3I). To mimic the heterozygous situation seen in patients with *PHOX2B* mutations, we repeated these experiments in the setting of *phox2b* MO knockdown. In this context, overexpression of *K155X* and *676delG* led to an even more striking reduction and, in some embryos, to an almost complete absence of *th* and *dbh* expression in the SCG (Figure 3O, 3P). These results suggest that the block in differentiation imposed by the *676delG* and *K155X* mutations cannot be rescued by the expression of endogenous wild-type *phox2b*; rather, these variants appear to function dominant-negatively.

### *phox2b* deficiency prevents retinoic acid-induced sympathetic progenitor cell differentiation

**Figure 4:**
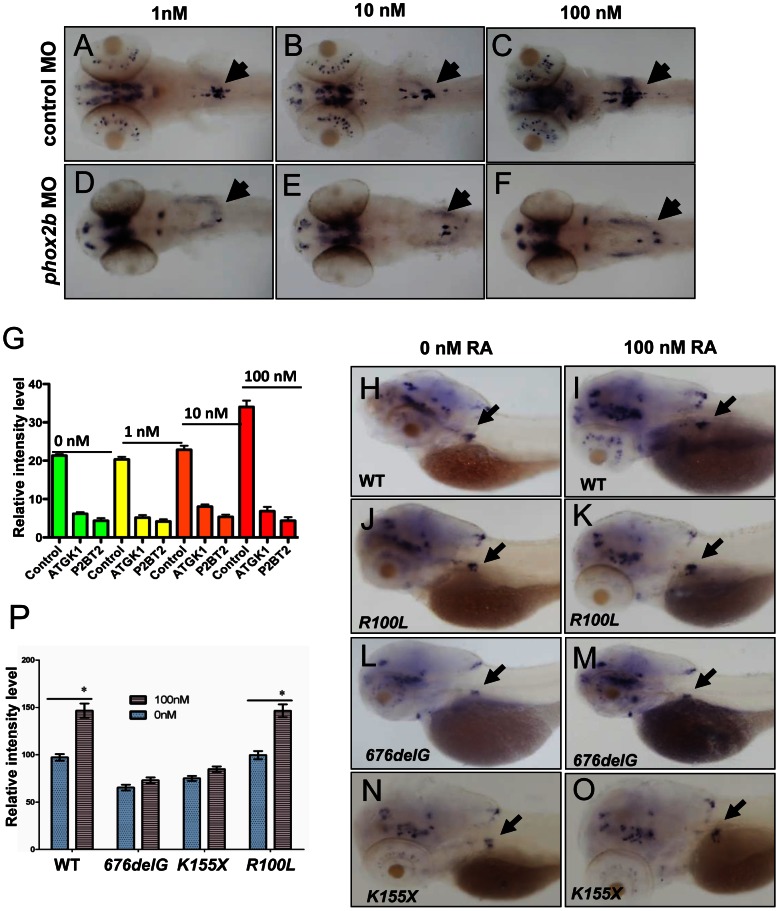
Impaired differentiation in the SCG due to *phox2b* deficiency is not rescued by retinoic acid. (A–F) Dorsal views of 3-dpf embryos injected with *phox2b* MO (D–F) or mismatched control MO (A–C) and treated with increasing concentrations of 13–*cis* retinoic acid (RA). (G) Relative intensity measurements of *th* expression in the SCG of embryos injected with various MOs and treated with different concentrations of RA. ATGK1, translation-blocking *phox2b* MO; P2BT2, splice blocking *phox2b* MO. (H–O) Whole-mount ISH of 3-dpf embryos in which the specified RNAs were overexpressed and analyzed for *th* expression following exposure to RA. Capped mRNA (100 ng/µl) for wild-type (WT) human *PHOX2B*, and the *R100L*, *676delG*, *K155X* mutations were injected into embryos at the one-cell stage. (P) Quantification of the relative intensity of *th* staining in the embryos depicted in H–O. Data are presented as means ± SD (^*^P<0.05; n = 10 per group).

*PHOX2B* complements exogenous differentiating agents such as retinoic acid (RA) by promoting cellular differentiation in vitro [36]. To determine whether *phox2b* loss might obviate the induction of differentiation by RA, we treated control and *phox2b* MO embryos with various concentrations of 13-*cis*-retinoic acid, which is commonly used in the treatment of patients with neuroblastoma (Figure 4A–4F). Control-injected embryos showed an increase in *th* expression in the SCG after RA treatment (Figure 4A–4C, 4G), which was not apparent in the SCG of the *phox2b* morphant embryos (Figure 4D–4F, 4G). A similar impairment in RA-induced differentiation was observed after expression of the *676delG* variant and, to a lesser extent, the *K155X* variant (Figure 4H–4P). Similar effects in *dbh* expression were also seen (data not shown). Together, these findings reinforce the strict requirement for Phox2b in the differentiation of sympathetic neuronal progenitors and suggest that loss of this transcription factor cannot be overcome with RA treatment.

### *phox2b* deficiency induces distinct effects on genes involved in PSNS differentiation

**Figure 5:**
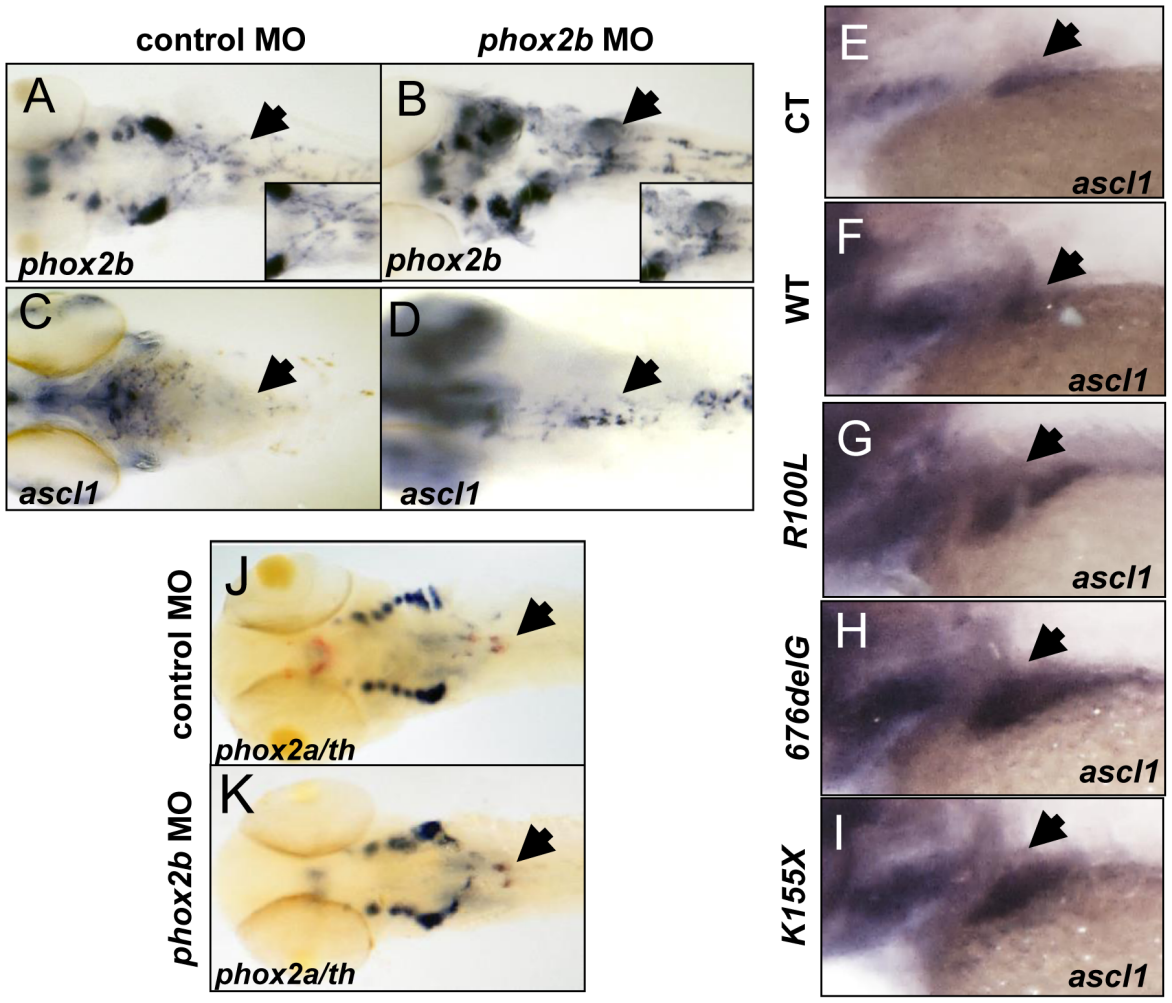
Deficiency of Phox2b protein due to either MO knockdown or overexpression of *PHOX2B* variants leads to increased *phox2b* and *ascl1* RNA expression in the SCG. (A–D) Dorsal views (cranial to the left) of 4-dpf embryos expressing a *phox2b* ATG MO (B,D) or mismatched control MO (A,C), showing expression of *phox2b* (A, B) and *ascl1* (C, D) as determined by whole-mount ISH. Insets depict an enlarged view of the area of the SCG. (E–I) Area of the SCG is shown in 4-dpf embryos (lateral view, cranial to the left) in which capped mRNA (100 ng/µl) for wild-type (WT) human *PHOX2B* (F) and the indicated variants (G–I) was injected and ISH performed for *ascl1*. CT, control, water injected. (J, K) Whole-mount ISH in control vs. *phox2b* MO-injected embryos double labeled with *phox2a (blue)* and *th* (red) riboprobes. Arrows point to the SCG.

To assess the impact of decreased *phox2b* function on the transcription factors that mediate noradrenergic differentiation in the zebrafish, we analyzed their expression following MO knockdown. We observed that mRNA expression of *phox2b* itself was markedly increased despite the decrease in Phox2b protein induced by MO knockdown (Figure 5A, 5B; Figure S5A, S5B). During normal development in the zebrafish, *phox2b* expression is first seen in sympathetic ganglia precursors at 36 hpf and, as the cells undergo differentiation, decreases to lower levels by 4 dpf (this study), corresponding to E10.5 to E13 in mice [6]. This increase in *phox2b* RNA expression on abrogation of the protein is consistent with studies that Phox2b regulates its own expression [31]. We also noted that expression of the zebrafish ortholog of *ASCL1*, *ascl1*, was strikingly increased in the SCG in *phox2b* morphants (Figure 5C, 5D; Figure S5C, S5D). *ascl1* is expressed only transiently in the SCG with maximal expression at 48 hpf, decreasing to less than 10% in 3-dpf embryos [18]. The increased expression of *ascl1* transcripts in the SCG of *phox2b* morphants suggests that Phox2b negatively regulates *ascl1* transcription as well. Similar to the effect seen with *phox2b* MO knockdown, overexpression of the neuroblastoma-associated variants also led to increased *ascl1* expression in the SCG, with the *676delG* and *K155X* mutants inducing higher *ascl1* expression than did *R100L* (Figure 5E–5I). *Phox2a*, a homologue of *Phox2b*, that is expressed downstream of both *Ascl1* and *Phoxb* during murine sympathetic neural development [13], [17] was unchanged in *phox2b* morphant embryos (Figure 5J, 5K). Finally, expression of *hand2* [19], [43], *gata3* [20], [44] and *tfap2a* [21], [22], three other transcriptional regulators implicated in the control of sympathetic neuronal differentiation [10], [18] was decreased on *phox2b* knockdown (Figure S5E–J). Overexpression of the *676delG* and *K155X* variants also led to a decrease in *gata3* and *tfap2a* expression (Figure S5K–P). Together, these results suggest that these genes are regulated by *phox2b* during sympathetic neuronal differentiation and are affected by perturbations in its function.

### *phox2b* deficiency leads to an increase in progenitor cells in the SCG

**Figure 6:**
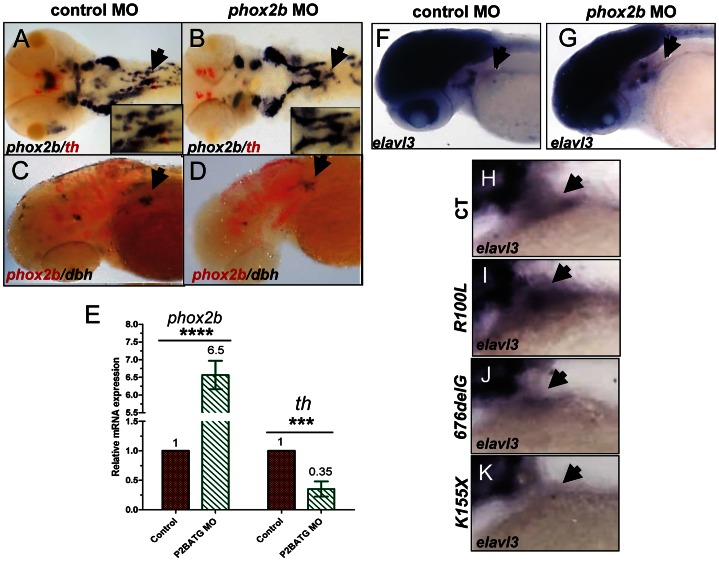
Phox2b deficiency causes arrest of SCG cells at an undifferentiated stage. (A,B) Dorsal views of ISH with FITC-labeled *th* (red) and digoxigenin-labeled *phox2b* (blue) riboprobes on 4-dpf embryos in which *phox2b* expression was abrogated by MO knockdown. Arrows point to the SCG. Insets show enlarged views of the SCG. (C, D) Lateral views of ISH with FITC-labeled *phox2b* (red) and digoxigenin-labeled *dbh* (blue) riboprobes in MO-injected (D) and control (C) embryos. (E) Quantitative real time-PCR analysis comparing *phox2b* mRNA expression levels in control vs. *phox2b* MO-injected (P2BATG MO) embryos. Data are presented as means ± SD (^****^P<0.0001, ^***^P<0.001; n = 15 per group). (F,G) Whole-mount ISH for *elavl3* in the area of the SCG (arrow) in *phox2b* MO-injected (G) compared to control MO-injected embryos at 4-dpf (F). (H–K) Lateral views of the SCG in 4-dpf embryos overexpressing the indicated RNAs analyzed for *elavl3* expression by whole-mount ISH.

We surmised that reduced expression of the *th* and *dbh* noradrenergic differentiation markers in the SCG of *phox2b* morphants might reflect either apoptosis of terminally differentiated sympathetic neurons or the failure of precursors to differentiate. There was no evidence of an increase in apoptosis in the SCG as determined by acridine orange staining of *phox2b* MO-injected embryos (data not shown). However, simultaneous analysis of *th* and *phox2b* expression by double labeling of the *phox2b* morphants showed an increase in *phox2b* expression with a concomitant decrease in *th* expression in the SCG compared to stage-matched control MO-injected embryos (Figures 6A, 6B). Analysis of these embryos using qRT-PCR confirmed the presence of significantly increased *phox2b* expression in the face of decreased *th* (Figure 6E). The same results were obtained when double labeling was performed with *dbh* and *phox2b*, the decrease in *dbh*-expressing cells being accompanied by increased expression of *phox2b* in morphants compared to controls (Figures 6C, 6D). We were unable to test whether this was also true in embryos in which *PHOX2B* variants were overexpressed because the high level of sequence homology between human and zebrafish *phox2b* made it difficult to interpret the results. To confirm that these *phox2b-* and *ascl1-*expressing SCG cells were arrested early in differentiation, we analyzed the expression of *elavl3* (*HuC*), the well-characterized, evolutionarily conserved neural differentiation marker that is normally only detected in mature postmitotic neurons and has been used to demonstrate neuronal differentiation in the zebrafish [41], [45]–[47] (Figures 6F–6K). We observed a decrease in *elavl3* expression in the *phox2b* morphants compared to controls, with the extent of reduction comparable to that of *th* and *dbh*, suggesting that the SCG is populated mainly by undifferentiated cells. Overexpression of the *676delG* and *K155X* mutants, but not *R100L*, also led to a decrease in *elavl3* expression in the SCG of these embryos (Figures 6H–K). Together, these data suggest that a decrease in Phox2b levels, either through MO knockdown or overexpression of dominant-negative mutations, blocks sympathetic cells at a progenitor stage marked by high *phox2b* and *ascl1* expression. Such cells cannot proceed to the next developmental stage, involving *hand2*, *gata3* and *tfap2a*, and therefore cannot undergo terminal PSNS differentiation characterized by the expression of *th* and *dbh*.

### *ascl1* is not essential for differentiation of SCG cells in zebrafish

**Figure 7:**
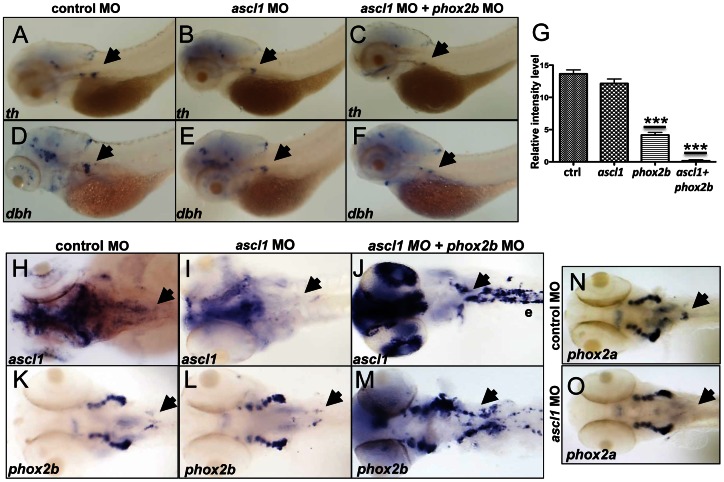
*phox2b*, but not *ascl1*, is indispensable for sympathetic neuronal differentiation. (A–F) Whole-mount ISH of 3-dpf embryos for *th* and *dbh* following *ascl1* MO knockdown. Lateral views depict a minimal decrease in expression of *th* (A,B) and *dbh* (D,E) in *ascl1* MO-injected embryos compared to control MO-injected (A,D) embryos. Simultaneous knockdown of both *ascl1* and *phox2b* led to a significant decrease in expression of *th* and *dbh* in the SCG (C,F). (G) Relative intensity levels of *dbh*-staining in the SCG of embryos expressing *ascl1* MO, *phox2b* MO or the combination of the two. Data are presented as means ± SEM (^***^P<0.001; n = 15 per group). (H–M) Whole-mount ISH for expression of *ascl* (H–J) and *phox2b* (K–M) in embryos in which *ascl1* expression was abrogated by MO knockdown, either singly (I, L) or in combination with *phox2b* (J, M). (N,O) Whole-mount ISH for *phox2a* expression in 4-dpf embryos in which *ascl1* expression was abrogated by MO knockdown. Arrows point to region of SCG.

During noradrenergic development in the chick embryo, *Phox2b* and *Ascl1* are expressed together in response to BMP signaling [44], [48]; in fact, elimination of *Ascl1* has also been reported to cause impaired sympathetic differentiation [14], [16]. To determine whether *ascl1* is as critical as *phox2b* to PSNS differentiation in the zebrafish, we studied the effects of *ascl1* knockdown using a translation-blocking MO (Figure S6A; Figure 7A, 7B, 7C, 7E). Abrogation of *ascl1* alone led to only a marginal decrease in *th* and *dbh* expression in the SCG (Figure 7B, 7E, 7G), while a striking decrease in both *th* and *dbh* expression occurred with simultaneous knockdown of both *ascl1* and *phox2b*, similar to the result with *phox2b* knockdown alone (Figure 7C, 7F, 7G). Moreover, in contrast to the outcome of *phox2b* knockdown, expression of *hand2*, *gata3* and *tfap2a* was not affected in the *ascl1* morphants (Figure S6B–G). These findings identify Phox2b as the central driver of terminal differentiation of sympathetic neurons in the zebrafish model.

### *ascl1* does not regulate *phox2b* in zebrafish

Observations in murine models have revealed that PHOX2B and ASCL1 cross regulate each other [6], [14], [16], [49]. The fact that *ascl1* expression was significantly increased in the *phox2b* morphants suggested a regulatory effect of phox2b on *ascl1* (Figure 5C,5D). To determine if Ascl1 had the same effect on *phox2b* in the zebrafish model, we studied the effects of *ascl1* knockdown on *phox2b* expression. While causing a decrease in its own expression at both the RNA and protein levels (Figure 7H, 7I, Figure S6A), knockdown of *ascl1* had no effect on *phox2b* expression (Figure 7K, 7L). Similarly, overexpression of *ascl* RNA also did not affect *phox2b* expression (Figure S6H, S6I). However, knockdown of both genes simultaneously led to a striking increase in *ascl1* (Figure 7J) and *phox2b* (Figure 7M) mRNA expression in the SCG. These results suggest that although Phox2b is capable of regulating *ascl1* in the zebrafish model, the latter does not have any appreciable impact on *phox2b*. Rather, Ascl1 appears to primarily regulate *phox2a*. Unlike knockdown of *phox2b*, which did not affect *phox2a* expression, we observed a significant reduction in *phox2a* expression in the SCG following *ascl1* knockdown (Figure 7N, 7O). These results are consistent with the finding that Ascl1 is required for the expression of *Phox2a* in sympathetic and parasympathetic murine ganglionic anlagen [14], [16], with *Phox2b* assuming a less important role.

## Discussion

**Figure 8:**
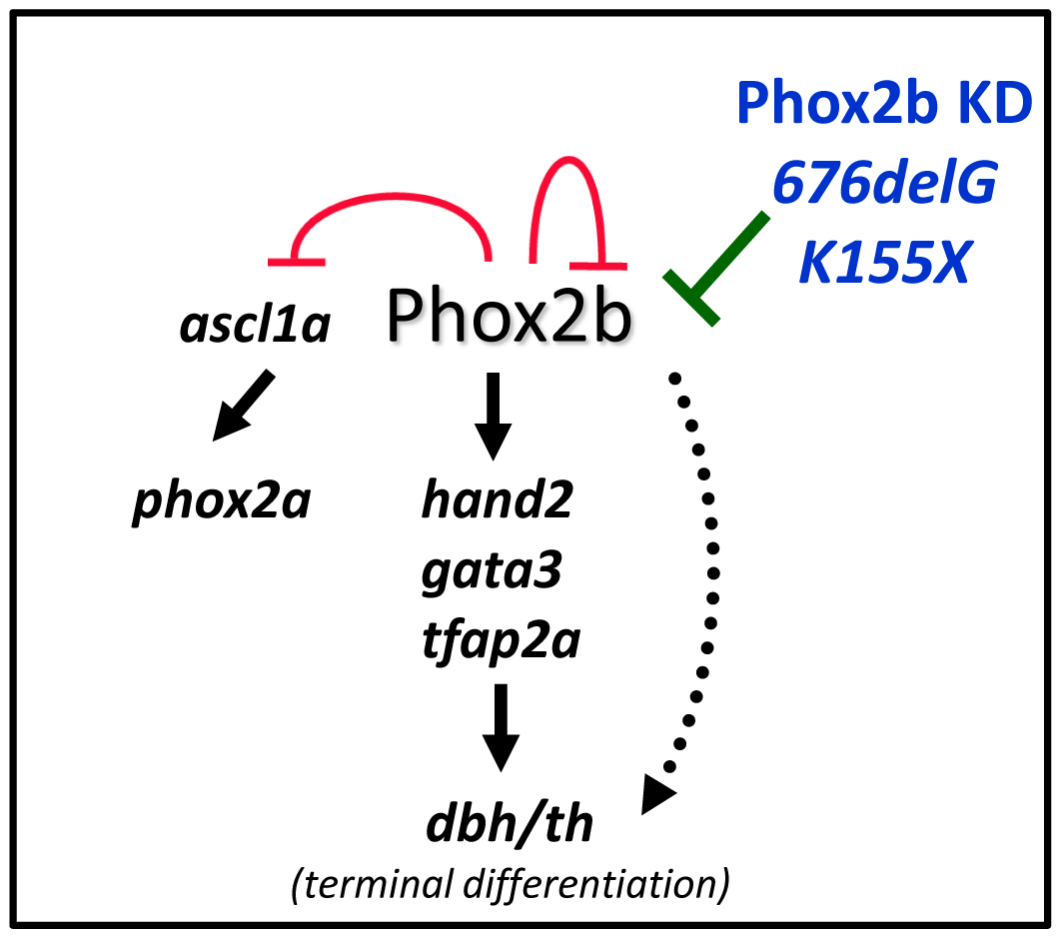
Schematic representation of the effect of aberrant Phox2b on sympathetic neuronal development in the zebrafish model. Phox2b is the master regulator of a differentiation cascade involving *Hand2*, *Gata2/3* and *Tfap2a* that ultimately leads to terminal differentiation of neuron progenitors, marked by *dbh* and *th* expression. Phox2b regulates itself as well as *ascl1a* and can directly activate *dbh*. A decreased dosage of the *phox2b* gene, either by allelic deletion (Phox2b KD) or by dominant-negative mutations (*676delG* or *K155X*) can compromise normal Phox2b function so that the cells are unable to progress through the various developmental stages and instead remain in an undifferentiated state.

In this study, we relied on a zebrafish model of PSNS development to demonstrate that a reduction in *phox2b* expression due to MO knockdown or overexpression of the neuroblastoma-associated *676delG* frameshift and *K155X* truncation variants of *PHOX2B* inhibits the terminal differentiation of sympathetic neuron progenitors (Figure 8). Our findings indicate that intact Phox2b function is essential for the normal development of cells in the sympathetic ganglia, and that in the context of *phox2b* deficiency, differentiation cannot be induced by exogenous agents.

In contrast to the remainder of the nervous system, where the onset of neuronal differentiation is coupled with cell cycle exit, an increase in the number of sympathetic neurons during development reflects the proliferation of differentiated sympathetic neurons rather than the proliferation and subsequent differentiation of neuronal progenitors [50], [51]. Indeed, studies in the chick embryo have revealed that the vast majority of postmitotic sympathetic neurons are generated through the proliferation of already differentiated neurons [50]–[53]. An alteration in Phox2b function in the zebrafish, by either MO knockdown or overexpression of certain neuroblastoma-associated variants, led to a block in the differentiation of sympathetic progenitor cells. First, there was a large increase in *phox2b* transcript-expressing cells at the expense of *th-* and *dbh*-expressing cells in the SCG (Figure 6). Second, these *phox2b*-expressing cells were unable to proceed to subsequent differentiation steps, even when stimulated with RA, whose signaling can induce noradrenergic differentiation in the zebrafish (Figure 4) [21]. Third, in addition to decreased *th* and *dbh* expression, the SCG cells also showed reduced expression of the mature neuronal marker *elavl3* (Figure 6).

In our study, specific types of *PHOX2B* variants exhibited distinct effects on sympathetic neuronal differentiation, with the *R100L* missense mutation lacking an effect on differentiation, while the *676delG* frameshift and *K155X* truncation mutations exhibited impaired differentiation, an effect that was all the more prominent with simultaneous MO knockdown. Findings similar to ours were reported by Reiff et al (2010) on overexpression of the *K155X* mutation in primary chick sympathetic neuron cultures, although in this system the *676delG* variant lacked any apparent effect on the expression of terminal differentiation markers [30]. In agreement with our data, another group reported resistance to differentiation with RA in human neuroblastoma cells engineered to express the *676delG* variant (in the presence of endogenous *PHOX2B*) [36]. The *R100L* homeodomain variant, on the other hand, did not appear to affect sympathetic neuronal differentiation in the zebrafish, similar to findings in the above mentioned study in avian sympathetic neurons, where this mutant elicited increased cell proliferation only [30].

The basis for the varied effects of distinct mutations on sympathetic neuronal differentiation is not entirely clear. To some extent, they reflect intrinsic differences between the in vitro and in vivo models in which the variants were tested, as well as different degrees of forced or blocked expression in target cells. Ultimately, however, the impact of *PHOX2B* mutants on the differentiation of immature sympathetic neurons depends on the protein structures that are modified. The *R100L* missense variant, for example, is defined by a minimal change in its homeodomain, whereas both *676delG* and *K155X* possess modifications due to an altered reading frame or deletion of critical coding sequences within the C-terminus, respectively, either of which can lead to the disruption of essential protein-protein interactions or, in some instances, to novel interactions not seen with the WT protein. Indeed, misfolding and oligomerization have been demonstrated with frameshift mutations of *PHOX2B* and cellular mislocalization and cytoplasmic aggregation with truncated variants [54]. The *693del8* frameshift mutant, for example, is thought to interact with proteins that do not bind to the WT protein [38]. Similarly, we have noted that the *676delG* and *K155X v*ariants do not bind to certain proteins recognized by WT *PHOX2B*, resulting in impaired neuroblastoma cell differentiation (George et al, unpublished observations). Thus, it is not surprising that the *K155X* truncation mutation, unlike the R100L missense change, generates a protein that not only stimulates sympathetic neuron proliferation [30], but also has a dominant–negative inhibitory effect on cell differentiation.

Furthermore, since both frameshift and truncation changes in the *PHOX2B* gene appear to generate stable proteins that lack the transactivation potential of WT *PHOX2B*, [30], [36], [37], [55] their contribution to neuroblastoma predisposition could be twofold. By interacting with different modifier proteins, these mutants could preserve the ability of PHOX2B to suppress cellular proliferation while abolishing its pro-differentiation regulatory effects. At the same time, as has been shown previously [30] these transactivation–impaired variants likely compete with intact *PHOX2B* for critical promoter sequences on neuronal differentiation-linked target genes, resulting in a dominant-negative repressive effect on their expression. This hypothesis is supported by the failure of the WT *phox2b to* rescue the arrested differentiation of sympathetic neuronal progenitors expressing either the *676delG* or *K155X* variants.

The gene dosage of *PHOX2B* also appears to have played a role in determining the disease phenotype in our study. Indeed, fish with MO knockdown of *phox2b* but no mutations or deletions consistently showed arrested differentiation of sympathetic neuronal progenitors. This result would account for a neuroblastoma case recently reported by Jennings et al [35] in which a heterozygous deletion of *PHOX2B* was associated with both CCHS and a neural crest tumor. Thus, *PHOX2B* deficiency due to whole-allele deletion should be considered another mechanism whereby individuals might acquire a predisposition to neuroblastoma. The precise oncogenic mechanism of *PHOX2B* deficiency and its associated phenotypes will require additional study, but it seems likely that dosage reduction beyond 50% (sub-haploinsufficiency) may be required, as mice that were haploinsufficient for *Phox2b* did not develop tumors [56].

Neuroblastoma development in *TH-MYCN* transgenic mice begins with hyperplastic lesions in early postnatal sympathetic ganglia that are composed predominantly of *Phox2b*-positive but *Th*-negative neuronal progenitors [28]. This indicates a central role for aberrant PHOX2B regulation of immature sympathetic neurons in neuroblastoma predisposition. Our studies suggest a model (Figure 8) in which aberrant PHOX2B function through either allelic deletion or dominant-negative mutations promote the same endpoint: impaired differentiation of sympathetic neuronal progenitor cells while promoting the expansion of a population of undifferentiated cells likely to be susceptible to so-called second “hits” such as *MYCN* amplification.

## Materials and Methods

### Ethics statement

All experiments involving zebrafish conformed to the regulatory standards and guidelines of the Dana-Farber Cancer Institute (DFCI) and the Brigham and Women's Hospital (BWH) Institutional Animal Care and Use Committee.

### Fish husbandry and strains

Zebrafish were maintained at the DFCI and BWH zebrafish facilities under standard conditions [57]. Embryos were raised in E3 medium supplemented with 0.003% 1-phenyl-2-thiourea (PTU) at 28.5°C to allow visualization of internal structures. The p53^−/−^ mutant allele *tp53^M214K/M214K^* was kindly provided by A. Thomas Look [42]. *tp53^M214K/M214K^* and wild-type embryos of the AB strain were obtained by natural group mating. The embryos were staged according to established morphological criteria [57].

### Morpholino injections

Morpholinos (MOs) were obtained from GeneTools, LLC (Philomath, OR) based on the published GenBank sequences for *phox2b* and *ascl1* (*phox2b* P2BT2 splice donor: 5′-AAGTAAGCGGAGAATGTCCCACCTG; mismatched *phox2b* P2BT2 splice donor: AcTAAcCGGAcAATcTCCgACCTG; *phox2b* ATG: 5′-TATACATTGAAAAGGCTCAGTGGAG; mismatched *phox2b* ATG: TATAgATTcAAAAccCTCAcTGGAG; *ascl1* ATG: 5′- CCATCTTGGCGGTGATGTCCATTTC; mismatched *ascl1* ATG: CCATgTTGGCcGTcATcTCgATTTC). For knockdown of *phox2b*, two nonoverlapping MOs were used (ATGK1, designed to target the start site; and P2BT2, designed to block splicing at the *phox2b* exon 2-intron 2 boundary). Embryos at the one to two-cell stages were injected with up to 4 ng of MO diluted with 1× Danieau's solution with Phenol Red. Injected embryos were raised to 12, 24, 36, 72, 96 and 120 hpf and processed for in situ hybdridization. Generalized nonspecific necrosis in MO-injected and mismatched control MO-injected AB embryos was alleviated using the *tp53^M214K/M214K^* strain [42]. MO concentrations were optimized so that the lowest amount necessary to retain the normal phenotype while demonstrating effects of absent *phox2b* expression was used. The observed phenotypes were consistent between the ATG and splice MO. Care was taken to stage-match the embryos as much as possible with mismatched MO-injected controls so as to eliminate phenotypes due to developmental delay.

### Capped RNA overexpression

Human *PHOX2B* (a generous gift from K-S Kim) and the *PHOX2B* mutant *676delG* (kindly provided by J. Maris) were subcloned into the pCS2+ vector. Site directed mutagenesis was used to generate *PHOX2B* mutant constructs *R100L* and *K155X* using the Quickchange II Site-directed mutagenesis kit (Stratagene). *ascl1* was amplified from a whole embryo cDNA library and cloned into pCS2+. The SP6 Message machine kit (Ambion) was used to transcribe synthetic capped RNA. Embryos were injected with 50–100 pg of mRNA at the one- to two-cell stages. Injected embryos were raised at different time points ranging from 12 hpf to 4 dpf and fixed with 4% paraformaldehyde for in situ hybridization.

### Antisense probes

The following antisense RNA probes were generous gifts: *phox2b* and *phox2a* (S. Guo), *dbh* and *elavl3* (J-S Lee). Other probes (*th*, *ascl1*, *tfap2a*, *hand2*, *gata3*) were generated by amplifying the *Danio rerio* ORFs from a whole embryo cDNA library using PCR primers based on published GeneBank sequences. The PCR products were subcloned into the *EcoRI/XhoI* sites of pCS2+ vector. Following sequence verification, antisense riboprobes were generated by in vitro transcription with DIG RNA labeling kit Sp6/T7 (Roche).

### Whole-mount in situ hybridization and immunofluorescence

Embryos at different developmental stages were collected and fixed in 4% (w/v) paraformaldehyde (Sigma). Whole-mount in situ hybridization was performed as described [58]. Images of zebrafish embryos were taken using an Olympus SZX12 microscope and a digital camera.

### Intensity measurements

Intensity measurements were performed using Image-Pro Plus (IPP) software (MediaCybernetics, PA). Identical regions were selected using the rectangle tool set to a constant area. Mean optical density (MOD) and total per area (TPA) in boxed areas were used for pixel intensity. Differences between two groups were analyzed using Student's t-test.

### Retinoic acid treatment

At 2 or 3 dpf embryos injected with a control MO and *phox2b* MO, were subjected to 13-cis retinoic acid treatment for 24 hrs at three concentrations 1 nM, 10 nM, and 100 nM diluted in PTU egg water. DMSO was used as a control. Embryos were then fixed and gene expression was analyzed using in-situ hybridization.

### RT-PCR

Embryos were homogenized in TRIzol reagent by passing through a 22G needle. Total RNA was extracted as described in the manufacturer's instructions (Invitrogen). Primer pairs encompassing the full-length *phox2b* gene were designed and used to amplify *phox2b* in the MO-injected embryos with the Superscript First strand synthesis kit (Invitrogen). 50 pg of 1^st^ strand cDNA was used to amplify *phox2b* using the Expand High Fidelity Plus PCR system (Roche).

### Western blotting

Immunoblotting was performed according to standard methods. The following antibodies were used: anti-PHOX2B (Santa Cruz, N14 cat# sc-48627), anti-ASCL1 (Santa Cruz, sc-28688 ASCL1-H56). Anti-beta tubulin antibody (Abcam 6046-100) was used as a loading control.

## Supporting Information

Figure S1 Zebrafish *phox2b* is highly homologous to human PHOX2B. (A) RT-PCR analysis of *phox2b* RNA expression in whole embryos at the indicated time points. (B) Western blot analysis of zebrafish Phox2b expression. (C) Phylogenetic tree depicting high conservation of *phox2b* among species. (D). Protein sequence alignment showing complete conservation of the homeodomain (HD, boxed) and the first polyalanine repeat (Ala 1) between human and zebrafish PHOX2B. The second polyalanine repeat, Ala 2, is absent in the zebrafish.
(TIF)

Figure S2 Abrogation of Phox2B expression by morpholino knockdown. (A) Schema of the genomic structure of the *phox2b* gene depicting the two morpholino (MO) sequences, a translation-blocking morpholino, MO^ATG^, and a splice-blocking morpholino, MO^splice^, targeted to the splice junction of the second exon. Exons are numbered 1, 2 and 3. The shaded boxes indicate the homeodomain. (B) Western analysis (left panel) of Phox2b expression in wild-type uninjected (WT), mismatched control MO-injected (ctrl MO) and *phox2b* MO-injected (MO^ATG^) embryos at the indicated time points. RT-PCR analysis (right panel), of 3-dpf embryos injected with either mismatched control MO (mm MO^splice^) or *phox2b* splice MO (MO^splice^) depicting a larger transcript (in addition to the WT *phox2b*) in the splice MO-injected embryos. Sequencing of the larger transcript revealed the inclusion of the second intron as shown in the accompanying schema. This transcript contained a stop codon (*) 40 bp into the second intron, leading to a truncated protein lacking the third exon. (C) The use of tp53^−^null embryos leads to a reduction in head necrosis and nonspecific morphological changes due to MO injection. Phenotypes of AB embryos show head necrosis and tail deformities (arrows) at 24 hpf and at 3 dpf after injection with Phox2b translation blocking (MO^ATG^), splice blocking (MO^Splice^) and to a lesser extent, mismatched control MOs (mm MO^ATG^) compared with uninjected (U) embryos. These defects are minimized in the *tp53* homozygous null background (*tp53^M214K/M214K^*) animals at 24 hpf and more obviously during later stages.
(TIF)

Figure S3 Phox2b knockdown leads to a significant decrease in the expression of terminal differentiation markers in the SCG. Whole mount ISH of *th* and *dbh* in 3-dpf embryos in which *phox2b* was abrogated by either a translation-blocking MO (B, ATGK1) or a splice-blocking MO (E, P2BT2) compared with control-MO injected (mmATGK1 and mmP2BT2) animals (A, D). Rescue of decrease in *th* and *dbh* expression with co-injection of human *PHOX2B* RNA (100 pg/µl) with either the translation-blocking Phox2b MO (C, ATGK1) or the splice-blocking MO (F, P2BT2). Relative intensity levels of *th* (G) and *dbh* (H) expression. Data are presented as means ± SD (^*^P<0.05, ^**^P<0.01, ^***^P<0.001; n = 15 per group).
(TIF)

Figure S4 Schema of selected *PHOX2B* mutations reported in patients with neuroblastoma. Variants tested in this study are marked in bold. Mutations *R100L* and *R141G* [7] are both located within the homeodomain. The *676delG* mutation [5] causes a frameshift and a premature stop, producing a slightly smaller protein containing only the first polyalanine tract. *K155X* (A463T) [8] encodes a premature stop codon and is predicted to produce a truncated protein missing the third exon (which contains both polyalanine tracts). NB, neuroblastoma; HSCR, Hirschprung's disease; CCHS, congenital central hypoventilation syndrome.
(TIF)

Figure S5 Aberrant *phox2b* expression leads to differential expression of genes involved in development of the noradrenergic lineage in the SCG. (A–D) Whole mount ISH for *phox2b* (A,B) and *ascl1* (C,D) at 3-dpf in embryos injected with *phox2b* MO (B,D) or mismatched control MO (A, C). Lateral views are shown, head to the left. (E–J) Whole mount ISH for the indicated probes in 3-dpf embryos in which *phox2b* expression was abrogated by MO knockdown. (K–P) Whole mount ISH for *gata3* (K–M) and *tfap2a* (N–P) expression in 4-dpf embryos in which the indicated mRNAs were injected. CT, control mRNA.
(TIF)

Figure S6 Alteration of *ascl1* levels does not affect the expression of *phox2b* or of genes involved in sympathetic neuronal differentiation. (A) Western analysis of Ascl1 expression in 72-hpf embryos injected with either control-MO (MM) or *ascl1* MO (*ascl1* MO). (B–G) Whole mount ISH for *hand*, (B,C) *gata3* (D,E), and *tfap2a* expression in 4-dpf embryos in which *ascl1* expression was abrogated by MO knockdown. Arrows point to the area of the SCG. (H, I) Whole mount ISH for *phox2b* expression in 4-dpf embryos injected with either control or *ascl* capped mRNA (100 µg/µl).
(TIF)
