# Supplementary figures and images for: Distinct Neuroblastoma-associated Alterations of PHOX2B Impair Sympathetic Neuronal Differentiation in Zebrafish Models
Source: PLoS Genet. 2013 Jun 6;9(6):e1003533. doi: 10.1371/journal.pgen.1003533 (PMC3675015; doi:10.1371/journal.pgen.1003533)

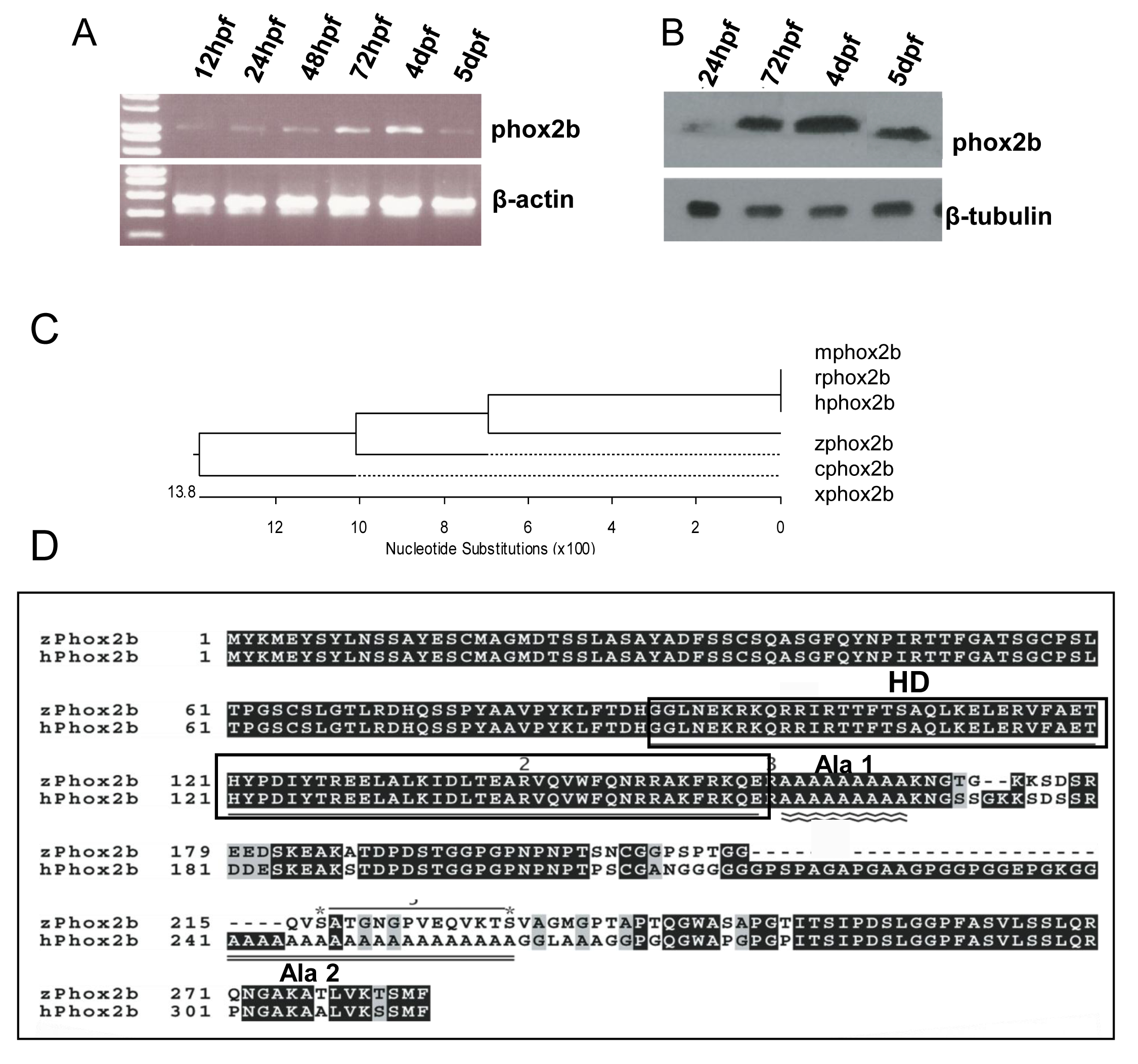

Supplement: Figure S1 — Zebrafish phox2b is highly homologous to human PHOX2B. (A) RT-PCR analysis of phox2b RNA expression in whole embryos at the indicated time points. (B) Western blot analysis of zebrafish Phox2b expression. (C) Phylogenetic tree depicting high conservation of phox2b among species. (D). Protein sequence alignment showing complete conservation of the homeodomain (HD, boxed) and the first polyalanine repeat (Ala 1) between human and zebrafish PHOX2B. The second polyalanine repeat, Ala 2, is absent in the zebrafish. (TIF) [file pgen.1003533.s001.tif]

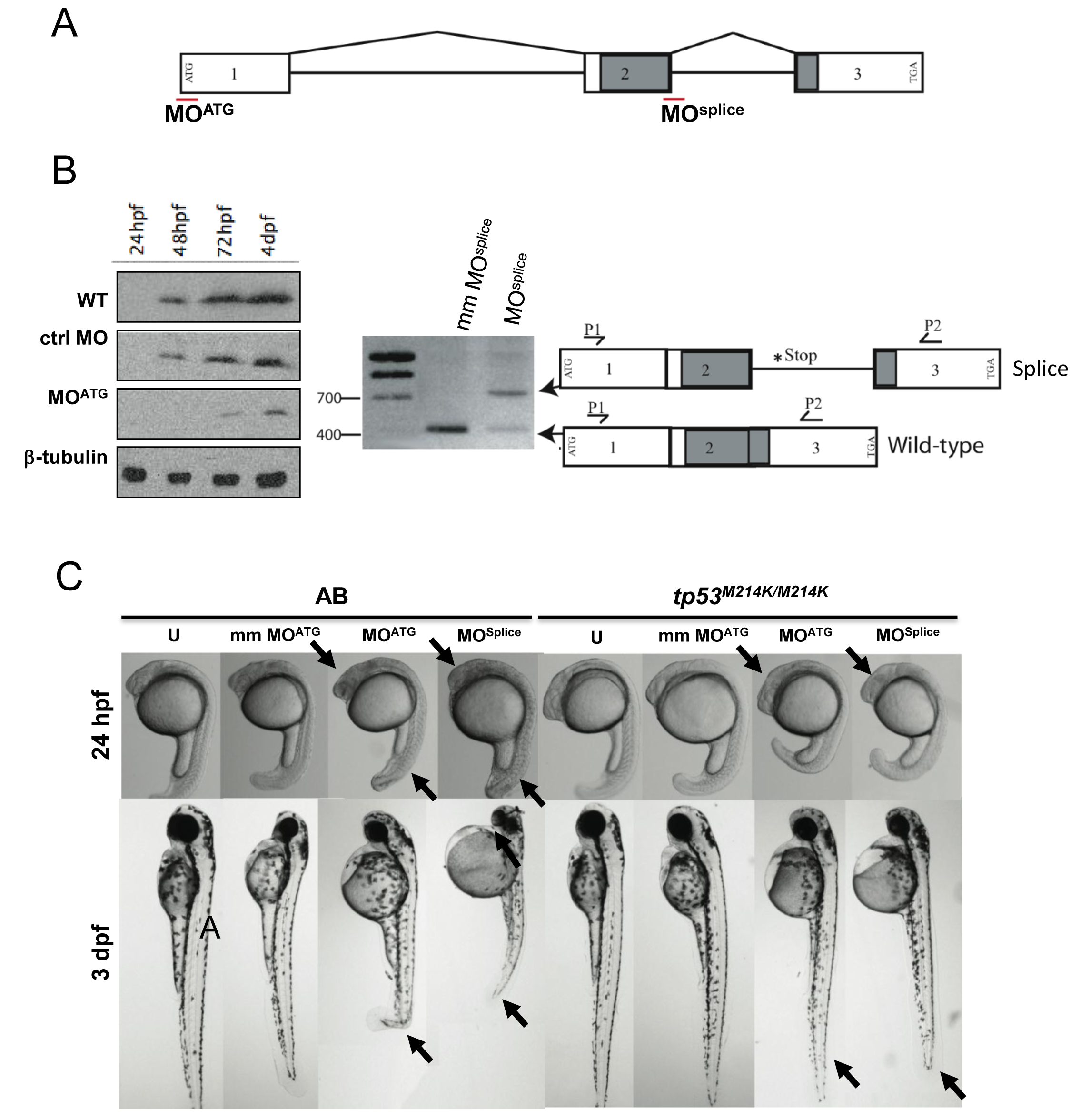

Supplement: Figure S2 — Abrogation of Phox2B expression by morpholino knockdown. (A) Schema of the genomic structure of the phox2b gene depicting the two morpholino (MO) sequences, a translation-blocking morpholino, MOATG, and a splice-blocking morpholino, MOsplice, targeted to the splice junction of the second exon. Exons are numbered 1, 2 and 3. The shaded boxes indicate the homeodomain. (B) Western analysis (left panel) of Phox2b expression in wild-type uninjected (WT), mismatched control MO-injected (ctrl MO) and phox2b MO-injected (MOATG) embryos at the indicated time points. RT-PCR analysis (right panel), of 3-dpf embryos injected with either mismatched control MO (mm MOsplice) or phox2b splice MO (MOsplice) depicting a larger transcript (in addition to the WT phox2b) in the splice MO-injected embryos. Sequencing of the larger transcript revealed the inclusion of the second intron as shown in the accompanying schema. This transcript contained a stop codon (*) 40 bp into the second intron, leading to a truncated protein lacking the third exon. (C) The use of tp53−null embryos leads to a reduction in head necrosis and nonspecific morphological changes due to MO injection. Phenotypes of AB embryos show head necrosis and tail deformities (arrows) at 24 hpf and at 3 dpf after injection with Phox2b translation blocking (MOATG), splice blocking (MOSplice) and to a lesser extent, mismatched control MOs (mm MOATG) compared with uninjected (U) embryos. These defects are minimized in the tp53 homozygous null background (tp53M214K/M214K) animals at 24 hpf and more obviously during later stages. (TIF) [file pgen.1003533.s002.tif]

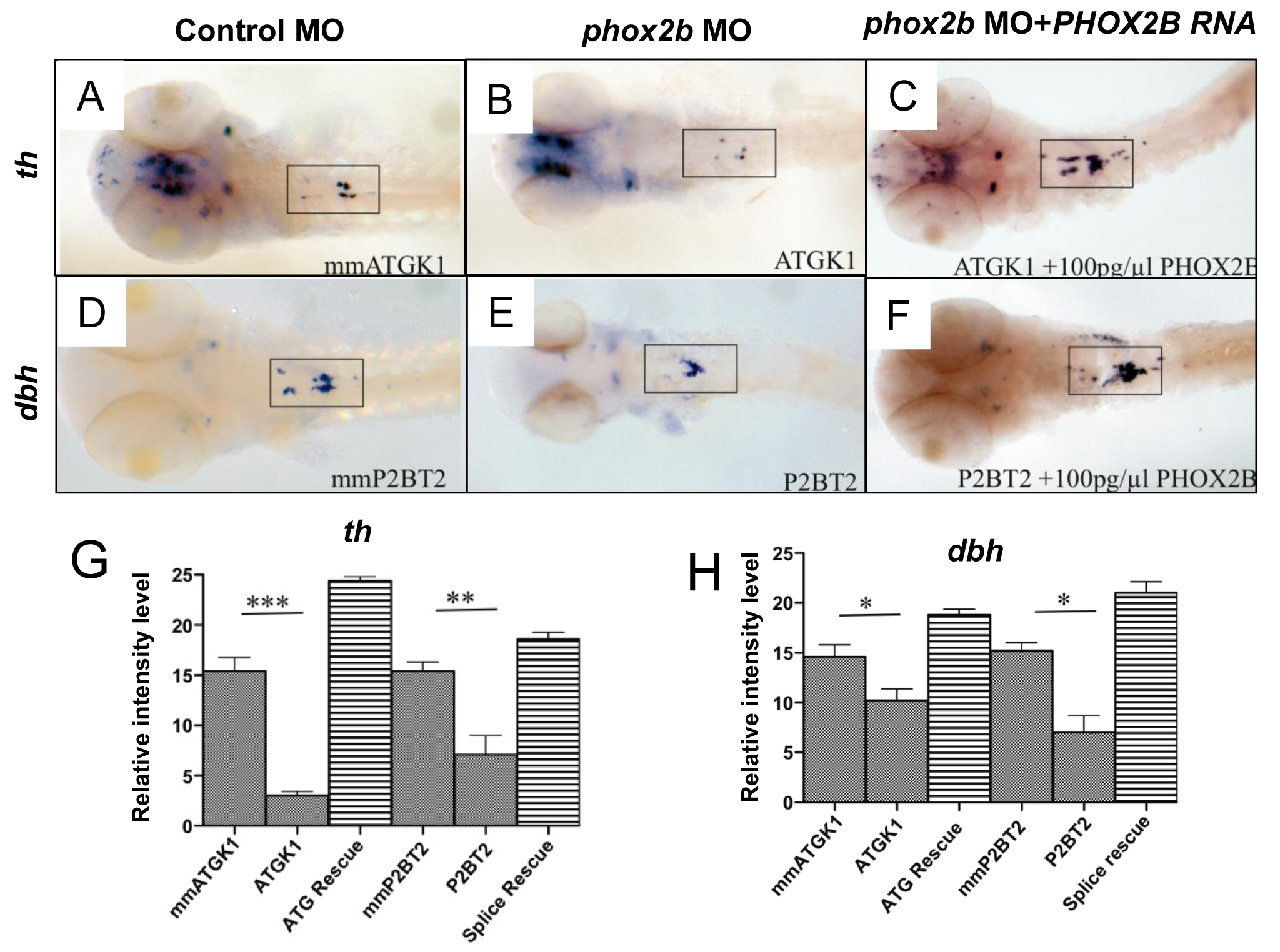

Supplement: Figure S3 — Phox2b knockdown leads to a significant decrease in the expression of terminal differentiation markers in the SCG. Whole mount ISH of th and dbh in 3-dpf embryos in which phox2b was abrogated by either a translation-blocking MO (B, ATGK1) or a splice-blocking MO (E, P2BT2) compared with control-MO injected (mmATGK1 and mmP2BT2) animals (A, D). Rescue of decrease in th and dbh expression with co-injection of human PHOX2B RNA (100 pg/µl) with either the translation-blocking Phox2b MO (C, ATGK1) or the splice-blocking MO (F, P2BT2). Relative intensity levels of th (G) and dbh (H) expression. Data are presented as means ± SD (*P<0.05, **P<0.01, ***P<0.001; n = 15 per group). (TIF) [file pgen.1003533.s003.tif]

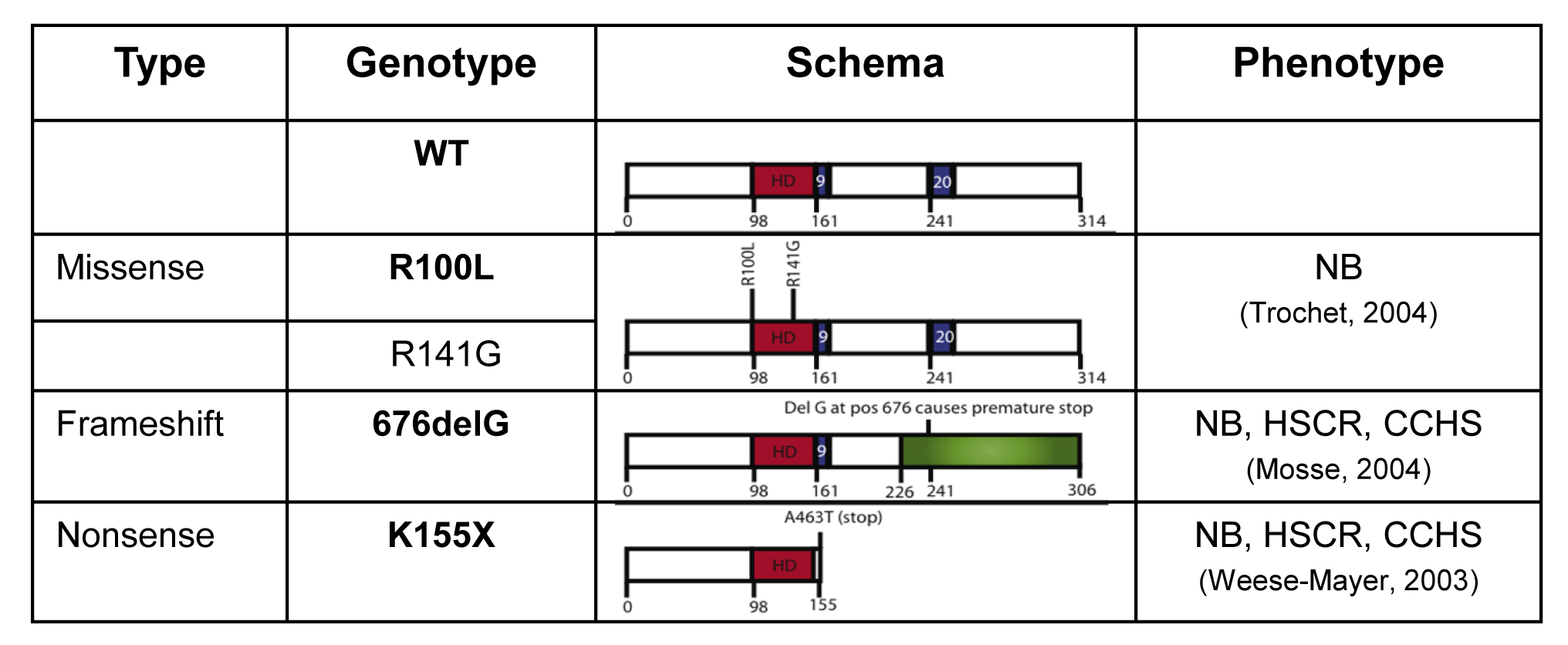

Supplement: Figure S4 — Schema of selected PHOX2B mutations reported in patients with neuroblastoma. Variants tested in this study are marked in bold. Mutations R100L and R141G [7] are both located within the homeodomain. The 676delG mutation [5] causes a frameshift and a premature stop, producing a slightly smaller protein containing only the first polyalanine tract. K155X (A463T) [8] encodes a premature stop codon and is predicted to produce a truncated protein missing the third exon (which contains both polyalanine tracts). NB, neuroblastoma; HSCR, Hirschprung's disease; CCHS, congenital central hypoventilation syndrome. (TIF) [file pgen.1003533.s004.tif]

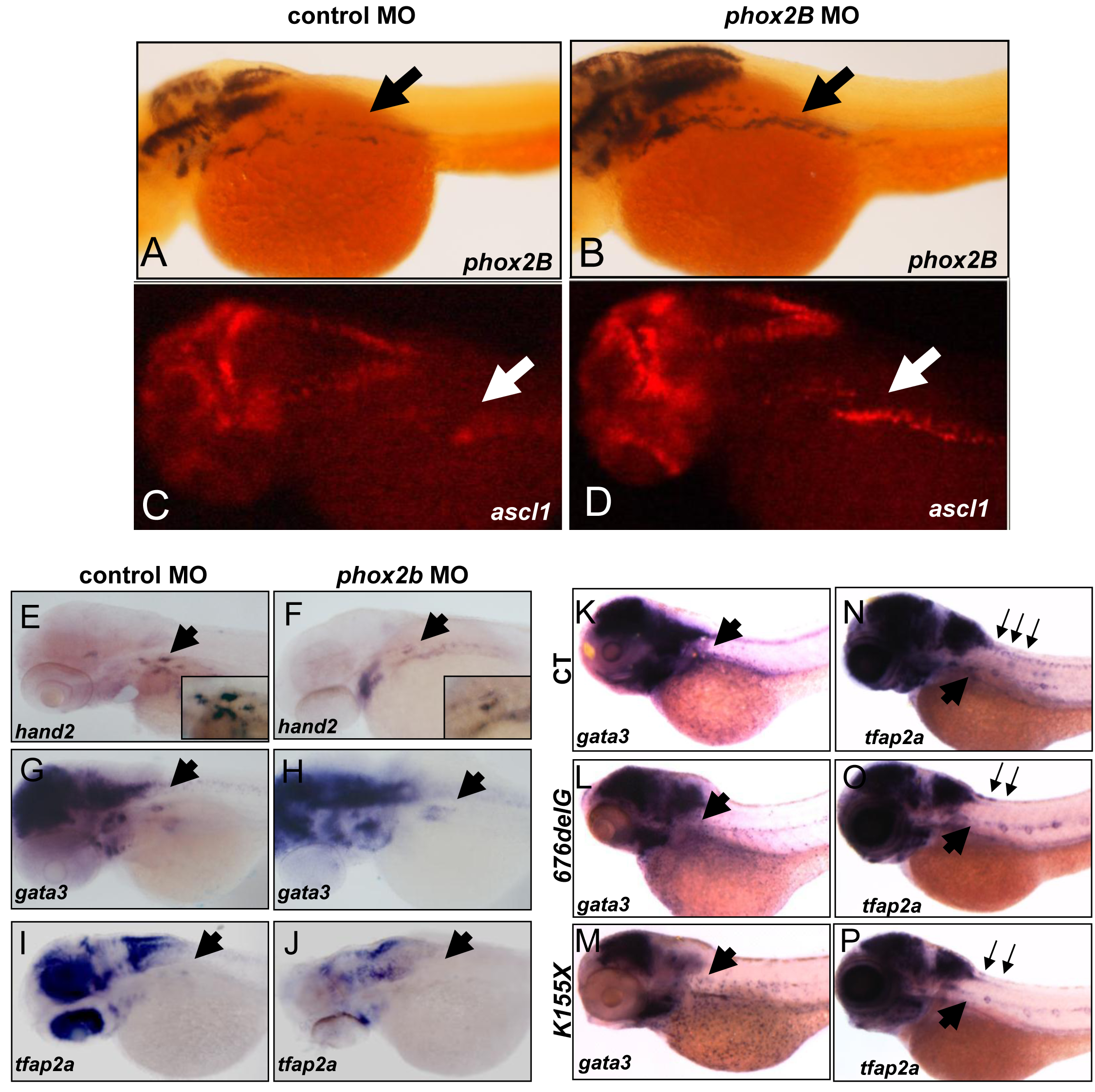

Supplement: Figure S5 — Aberrant phox2b expression leads to differential expression of genes involved in development of the noradrenergic lineage in the SCG. (A–D) Whole mount ISH for phox2b (A,B) and ascl1 (C,D) at 3-dpf in embryos injected with phox2b MO (B,D) or mismatched control MO (A, C). Lateral views are shown, head to the left. (E–J) Whole mount ISH for the indicated probes in 3-dpf embryos in which phox2b expression was abrogated by MO knockdown. (K–P) Whole mount ISH for gata3 (K–M) and tfap2a (N–P) expression in 4-dpf embryos in which the indicated mRNAs were injected. CT, control mRNA. (TIF) [file pgen.1003533.s005.tif]

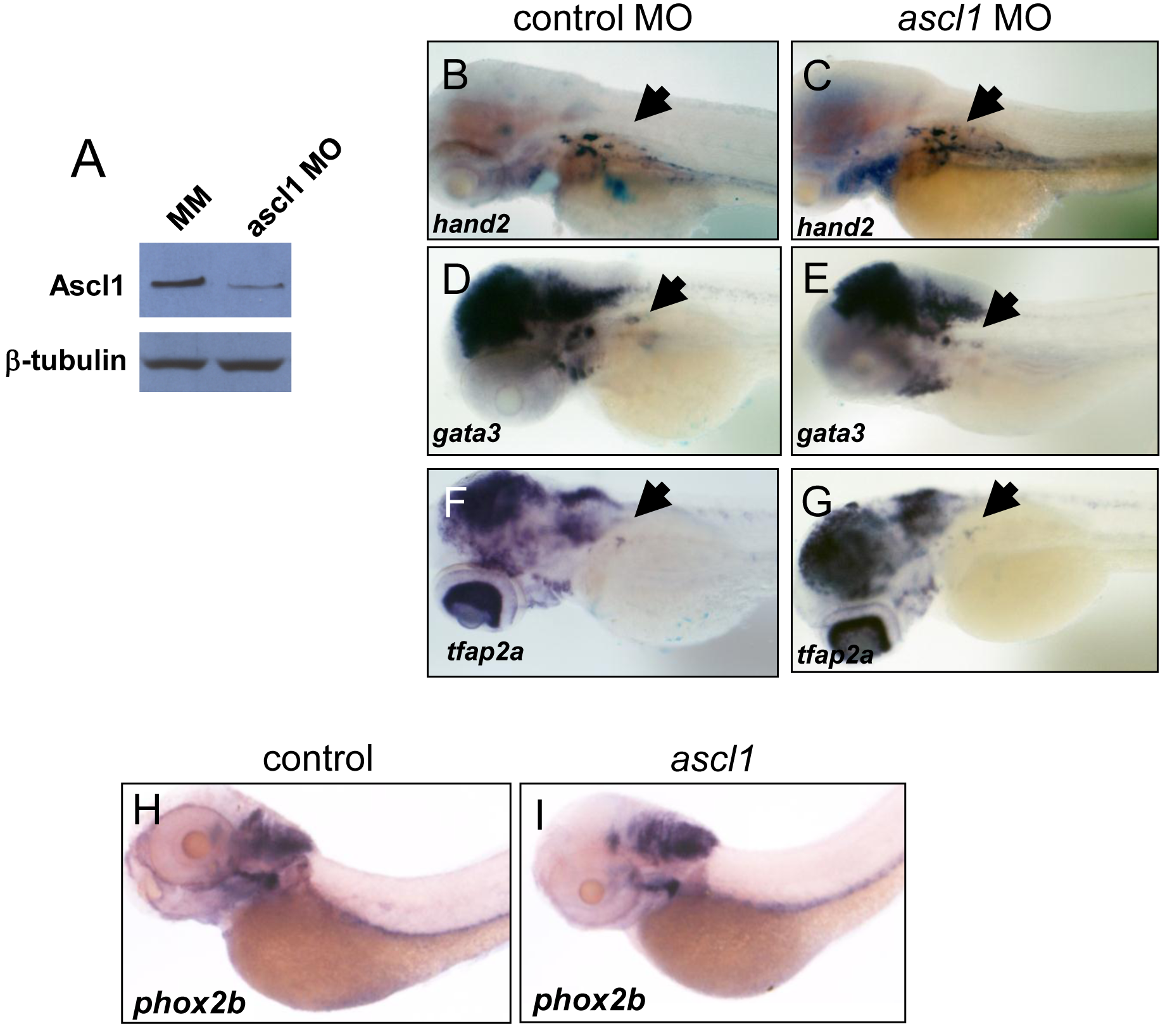

Supplement: Figure S6 — Alteration of ascl1 levels does not affect the expression of phox2b or of genes involved in sympathetic neuronal differentiation. (A) Western analysis of Ascl1 expression in 72-hpf embryos injected with either control-MO (MM) or ascl1 MO (ascl1 MO). (B–G) Whole mount ISH for hand, (B,C) gata3 (D,E), and tfap2a expression in 4-dpf embryos in which ascl1 expression was abrogated by MO knockdown. Arrows point to the area of the SCG. (H, I) Whole mount ISH for phox2b expression in 4-dpf embryos injected with either control or ascl capped mRNA (100 µg/µl). (TIF) [file pgen.1003533.s006.tif]
